# Characterization of a Type II-A CRISPR-Cas System in *Streptococcus mutans*

**DOI:** 10.1128/mSphere.00235-20

**Published:** 2020-06-24

**Authors:** Cas Mosterd, Sylvain Moineau

**Affiliations:** a Département de Biochimie, de Microbiologie, et de Bio-Informatique, Faculté des Sciences et de Génie, Université Laval, Québec City, Quebec, Canada; b Groupe de Recherche en Écologie Buccale, Faculté de Médecine Dentaire, Université Laval, Québec City, Quebec, Canada; c Félix d’Hérelle Reference Center for Bacterial Viruses, Faculté de Médecine Dentaire, Université Laval, Québec City, Quebec, Canada; University of California, Davis

**Keywords:** CRISPR, CRISPR-Cas, Cas9, *Streptococcus*, *mutans*, bacteriophages, phage resistance, plasmids, spacers

## Abstract

Streptococcus mutans and its virulent phages are important members of the human oral microbiota. S. mutans is also the primary causal agent of dental caries. To survive in this ecological niche, S. mutans must encode phage defense mechanisms, which include CRISPR-Cas systems. Here, we describe the CRISPR-Cas type II-A system of S. mutans strain P42S, which was found to display natural adaptation and interference activity in response to phage infection and plasmid transformation. Newly acquired spacers were integrated both at the 5′ end of the CRISPR locus and ectopically. In comparisons of the *cas* genes of P42S to those of other strains of S. mutans, *cas1*, *cas2*, and *csn2* appear to be highly conserved within the species. However, more diversity was observed with *cas9*. While the nuclease domains of S. mutans Cas9 (SmCas9) are conserved, the C terminus of the protein, including the protospacer adjacent motif (PAM) recognition domain, is less conserved. In support of these findings, we experimentally demonstrated that the PAMs associated with SmCas9 of strain P42S are NAA and NGAA. These PAMs are different from those previously reported for the CRISPR-Cas system of the model strain S. mutans UA159. This study illustrates the diversity of CRISPR-Cas type II-A systems that can be found within the same bacterial species.

**IMPORTANCE** CRISPR-Cas is one of the mechanisms used by bacteria to defend against viral predation. Increasing our knowledge of the biology and diversity of CRISPR-Cas systems will also improve our understanding of virus-bacterium interactions. As CRISPR-Cas systems acquiring novel immunities under laboratory conditions are rare, Streptococcus mutans strain P42S provides an alternative model to study the adaptation step, which is still the least understood step in CRISPR-Cas biology. Furthermore, the availability of a natural Cas9 protein recognizing an AT-rich PAM opens up new avenues for genome editing purposes.

## Abstract (precis)

CRISPR-Cas is one of the mechanisms used by bacteria to defend against viral predation. Increasing our knowledge of the biology and diversity of CRISPR-Cas systems will also improve our understanding of virus-bacterium interactions. As CRISPR-Cas systems acquiring novel immunities under laboratory conditions are rare, Streptococcus mutans strain P42S provides an alternative model to study the adaptation step, which is still the least understood step in CRISPR-Cas biology. Furthermore, the availability of a natural Cas9 protein recognizing an AT-rich PAM opens up new avenues for genome editing purposes.

## INTRODUCTION

More than 500 different bacterial species can be found in the human oral cavity, although very few of them can cause diseases (1). Streptococcus mutans is a Gram-positive bacterial species associated with dental caries, which is the most common oral disease. S. mutans metabolizes carbohydrates transiently passing through the mouth into various acids, including lactic acid (2). The resulting pH reduction demineralizes the hard tissue of the teeth, and the net loss of minerals over time leads to the formation of dental caries (3). S. mutans is also resistant to many environmental conditions (4), and its capacity to favor dental caries is likely due to a combination of its adhesion abilities, production of acids, and relative resistance to low pH (5).

Viruses are the most abundant biological entities in characterized Earth ecosystems, and globally they can infect all hosts, including bacteria (6). Bacterial viruses (phages) play a role in the regulation of bacterial populations, including in the oral microbiota (7). However, very few lytic S. mutans phages have been isolated and described in the literature (8, 9). For example, only the genomic sequences of phage M102 (9), M102AD (10), and ΦAPCM01 (11) are currently available in public databases.

To survive in phage-containing environments, bacteria have developed an impressive arsenal of antiphage mechanisms (12, 13). One of these numerous mechanisms is the CRISPR-Cas system. CRISPR (clustered regularly interspaced, short palindromic repeats) refers to a series of short palindromic nucleotide repeats interspaced with similarly sized spacers, and these arrays are found in less than half of bacteria (14), including in S. mutans (15). Along with a set of associated genes (*cas*), this system acts as a microbial adaptive immune system (16). To date, six different types of CRISPR-Cas systems have been identified and divided into several subtypes (17, 18). Although there are significant differences at the molecular level between the various types, they mostly function using a similar process.

First, short DNA protospacers from infecting phages (often from defective) (19) or plasmid sequences (20) are integrated into the CRISPR array as spacers in a process known as adaptation. An AT-rich sequence, called the leader sequence, is often found directly upstream of the CRISPR array and usually contains a promoter that allows transcription of the array into pre-crRNA (21–23). The pre-crRNA is then matured into small RNA molecules (24, 25). In type II systems, the small RNAs, also known as crRNA, are associated with Cas9 inside bacterial cells to recognize and cleave subsequent invading nucleic acids with sequences identical to that of the spacer (20). The DNA cutting activity observed with type II systems also requires the presence of a short nucleotide motif, called the protospacer adjacent motif (PAM), next to the target DNA (26, 27). This ability to target and to specifically cleave DNA has led to many applications, including in genome editing (28, 29). Another unique feature of type II systems is the requirement of tracrRNA. These are small RNA molecules that possess nucleotides of complementarity with the repeat regions of crRNAs. The complementarity will allow the formation of an RNA duplex which, in turn, facilitates crRNA maturation (25).

In a previous study (15), it was noted that 19 out of 27 (70%) examined S. mutans strains possessed a type II-A CRISPR-Cas system, which consists of the four genes *cas9*, *cas1*, *cas2*, and *csn2*. Moreover, 9 of the same 27 strains (33%) also possessed a type I-C CRISPR-Cas system, consisting of seven genes, which are *cas3*, *cas5*, *cas8c*, *cas7*, *cas4*, *cas1*, and *cas2*, while 15% of them (4 out of 27) possessed both types. Interestingly, 56% of the spacers (172 out of the 305 spacers) in the various CRISPR arrays of these S. mutans strains had homology to the genome of the virulent siphophage M102. Bioinformatic analyses also suggested that the type I-C system in the model S. mutans strain UA159 was inactive due to truncated *cas1* and *cas8c*. On the other hand, spacer acquisition was experimentally demonstrated for the type II-A system. Indeed, a 5′-end expansion of the CRISPR array was observed in bacteriophage-insensitive mutants (BIMs) isolated following exposure of the wild-type S. mutans strain UA159 to the phage M102 (15). Surprisingly, disruption of the type II CRISPR-Cas system did not restore phage sensitivity, suggesting the presence of additional antiviral systems (15, 30). Nonetheless, the PAM sequence recognized by the CRISPR-Cas system of strain UA159 was proposed to be 5′-NGG-3′ (15).

During the characterization of the virulent siphophage M102AD (10), it was demonstrated that it shares 90.8% identity at the nucleotide level with phage M102. Phage M102AD replicates on the host strain S. mutans P42S but not on S. mutans strain UA159. Here, we investigated the interactions between phage M102AD and its host, S. mutans P42S. We showed the presence of an active type II-A CRISPR-Cas system in S. mutans P42S following the characterization of BIMs obtained after a challenge with phage M102AD. However, bioinformatic analyses and functional studies indicated that this system recognizes a different PAM.

## RESULTS

### Analysis of the CRISPR-Cas systems of S. mutans P42S.

**FIG 1:**
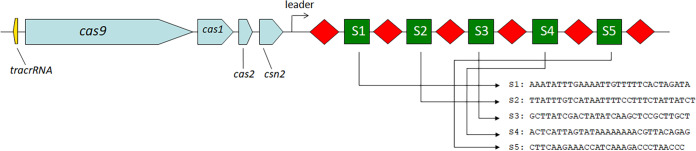
Type II-A CRISPR-Cas system of S. mutans P42S. Squences are from 5′ to 3′. The *tracrRNA* is in yellow, the *cas* genes are in blue, the leader is a black arrow, the repeats are represented as red diamonds, and the spacers are green squares.

Whole-genome sequencing of S. mutans P42S revealed one CRISPR locus, consisting of five spacers of 29 to 31 bp in length (Fig. 1), separated by the five identical 36-bp repeat sequences (5′-GTTTTAGAGCTGTGTTGTTTCGAATGGTTCCAAAAC-3′) and a terminal repeat that possessed a mutation at the final base pair (in boldface; 5′-GTTTTAGAGCTGTGTTGTTTCGAATGGTTCCAAAA**T**-3′). The same repeat sequence was observed in CRISPR arrays of other S. mutans genomes, including in UA159 (15). Of note, the third spacer in the CRISPR array of P42S had a stretch of 19 out of 20 bp identical to a segment of a gene of unknown function in the genome of phage M102AD. The other four spacers did not share any significant sequence identity with sequences in public databases, including spacers found in other S. mutans strains in the CRISPR database. Upstream of the CRISPR array, four *cas* genes associated with a type II-A system were found, namely, *cas9*, *cas1*, *cas2*, and *csn2* (Fig. 1). A *tracrRNA* was also found upstream of the *cas9* gene. No type I-C system was detected in this strain.

The whole-genome sequences are publicly available for 12 strains of S. mutans that possess a CRISPR array with the same repeat sequence as that found in S. mutans P42S. The CRISPR arrays in these strains contained between 3 and 70 spacers, with an average of 21 spacers. Remarkably, out of the 245 spacers detected in these strains, 89 of them partially matched the phage M102AD genome. Nine of these spacers matched 100% part of the phage M102AD genome. Only one (strain NCTC10920) did not possess any spacer with a level of identity to the genome of phage M102AD. The nucleotide sequences of the *cas* genes and deduced proteins of S. mutans P42S were compared to those found in the 12 strains mentioned above. The results are illustrated in Fig. S1 in the supplemental material. There was a high degree of identity with 11 of the 12 S. mutans strains in the *cas1* (96.3% to 100%), *cas2* (99.4% to 100%), and *csn2* (99.1% to 100%) sequences. Interestingly, more diversity was observed between the *cas9* sequences of these strains (82.5% to 99.9%), with the *cas9* sequence of P42S only being similar to that of strains Ingbritt (99.9%) and NCTC10832 (99.2%). Strain NCTC10920 was somewhat of an outlier, sharing the lowest identity to *cas9* (70.4%), *cas1* (89.9%), *cas2* (90.6%), and *csn2* (83.4%) of strain P42S.

10.1128/mSphere.00235-20.3 FIG S1 Comparison of *cas* genes (left) and Cas proteins (right) of nine S. mutans strains. Percent identity to the sequences found in S. mutans P42S is indicated. Download FIG S1, TIF file, 0.3 MB. Copyright © 2020 Mosterd and Moineau. 2020 Mosterd and Moineau This content is distributed under the terms of the Creative Commons Attribution 4.0 International license.

Similar observations were made with the deduced amino acid sequences of Cas1 (96.9% to 100% identity), Cas2 (98.1% to 100%), Csn2 (98.8% to 99.7%), and Cas9 (79.1% to 99.9%). The lowest identity of P42S Cas proteins again was with those of NCTC10920 (Cas9, 67.8%; Cas1, 95.1%; Cas2, 94.5%; and Csn2, 85.5%). Further investigations revealed that the difference between the Cas9 protein sequences mostly lies at the C terminus. In comparing the first 1,040 amino acids (out of the total of 1,345 to 1,370) of P42S Cas9 to the ones found in other S. mutans strains, the identity to 10 of the 12 other Cas9 proteins ranged between 95.1% and 99.9%. The sequence identity dropped to 88.4% for Cas9 of S. mutans strain GS-5 and 73.9% for NCTC10920. For the remaining 305 to 330 amino acids at the C terminus, the percent sequence identity between P42S Cas9 and the other S. mutans Cas9 proteins drops drastically. Sequence identity of the P42S Cas9 C terminus was 100% with strain Ingbritt and NCTC10832 but declined to between 46.6% and 47.2% with the other 10 strains.

**FIG 2:**
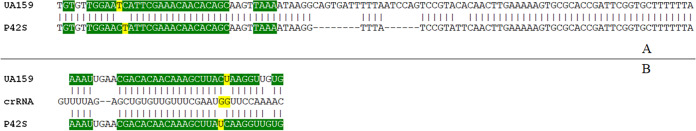
tracrRNA in S. mutans. (A) Comparison of predicted *tracrRNA* in S. mutans UA159 and P42S. Complementarity to crRNA is highlighted in green. (B) The anti-repeat region within the tracrRNA of S. mutans UA159 and P42S compared to crRNA.

A *tracrRNA* sequence was found upstream of the *cas9* gene of S. mutans P42S. The tracrRNA of S. mutans strain UA159 was previously estimated to be 107 nucleotides long (25). The tracrRNA of S. mutans P42S was estimated to be 93 nucleotides due to a deletion. However, within the anti-repeat region of the tracrRNA, there was almost full identity to the tracrRNA of S. mutans UA159, with the exception of two positions, where there were inversions. Still, there was a stretch of 25 nucleotides where 24 nucleotides of the tracrRNA matched the repeat sequence (Fig. 2). RNA duplex formation is necessary for the maturation of the pre-crRNA (25).

### BIM assays.

To determine if the type II-A CRISPR-Cas system of S. mutans P42S was functional, we performed BIM (bacteriophage-insensitive mutant) assays as described previously (16). Based on the random screening of 100 colonies, 20% of them acquired at least one new spacer after exposure to the virulent phage M102AD. In separate assays, when the phage lysate was exposed to UV light prior to the phage challenge assay (19), the fraction of BIMs that had acquired a new spacer increased to 68% (19 out of 28 colonies tested). Overall, most BIMs acquired one or two new spacers, but the acquisition of up to seven new spacers was observed in one BIM.

Newly acquired spacers were between 28 and 32 bp long, with 30 bp being the most frequent length. Out of the 168 unique acquired spacers, 114 were 30 bp (68%), 46 were 31 bp (27%), five were 32 bp (3%), two were 29 bp (1%), and one was 28 bp (<1%) long. The details of all 168 acquired spacers can be found in Table S1. Of these 168 acquired spacers, 148 of them (88%) were identical (100%) to a section of the phage M102AD genome. In addition, 10% (16 out of 168) of them had one or two nucleotide mismatches compared to the genome of M102AD. The remaining 2% (4 out of 168) had low (1 spacer) or no (3 spacers) identity to the genome of M102AD. Surprisingly, among these there was one spacer (spacer 101) that perfectly matched the genome of S. mutans P42S.

10.1128/mSphere.00235-20.1 TABLE S1 List of spacers acquired by different bacteriophage insensitive mutants of S. mutans. Mismatches to the M102AD genome are shown in italics in the protospacer sequence. Spacers that are not 100% identical to M102AD are indicated by an asterisk in the spacer column. Download Table S1, DOCX file, 0.02 MB. Copyright © 2020 Mosterd and Moineau. 2020 Mosterd and Moineau This content is distributed under the terms of the Creative Commons Attribution 4.0 International license.

In related type II-A systems, new spacers are typically integrated at the 5′ end of the CRISPR locus. The sequence upstream of the first repeat in the CRISPR array was shown to have an important role in specifying the integration site, since mutations in this leader sequence result in the integration of spacers within the CRISPR array (31). This ectopic spacer acquisition within the array following phage infection was observed in another *Streptococcus* species, namely, S. thermophilus (32). In S. mutans P42S, novel spacers were integrated at the 5′ end or within the CRISPR locus. Ectopic spacers in S. mutans P42S were acquired between spacers 4 and 5, except one, which was integrated between spacers 1 and 2. Ectopic spacer acquisition was broadly observed in BIMs that acquired multiple spacers, with at least one at the 5′ end of the CRISPR locus. Indeed, only two BIMs acquired a single spacer within the array. In both of these BIMs, the acquired spacer perfectly matched the genome of phage M102AD.

### Identification of the PAM.

**FIG 3:**
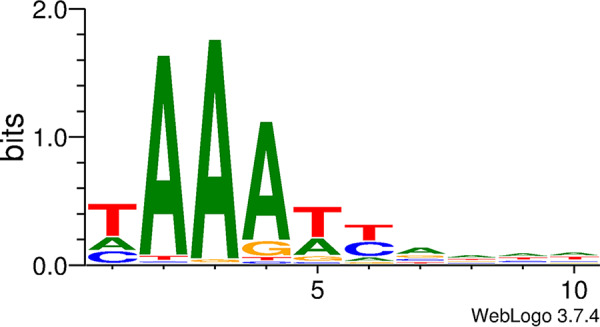
PAM downstream of protospacers.

Of the 168 newly acquired spacers, 165 could be mapped as protospacers on the phage M102AD genome (Table S1). The 10 bp upstream and downstream of each of the corresponding protospacers in the phage genome were analyzed for the presence of the protospacer adjacent motif (PAM) (Table 1). No particular motif was detected upstream of the protospacers. However, there was a clear preference for a 5′-NAAA-3′ motif downstream of the protospacers (Fig. 3). Indeed, 79% of the protospacers (131 out of 165) were flanked by the NAAA sequence. The adenine on position 4 was the least conserved. When considering only the NAA motif, the percentage of protospacers flanked by this sequence increased to 93% (153/165). The most commonly observed PAM was TAAAT, which was found next to 21% (35/165) of the protospacers. When considering only the spacers acquired at the 5′ end of CRISPR loci, 97% (85/88) of the protospacers were flanked by the trinucleotide NAA and 27% (24/88) were flanked by TAAAT. When analyzing just the spacers integrated at an ectopic position, 82% (19/23) of the protospacers were flanked by NAA and 13% (3/23) were flanked by TAAAT. Of interest, in BIMs that have acquired a single spacer, 100% (35/35) of the protospacers were flanked by NAA (35/35), while 35% (12/35) of them were flanked by TAAAT.

**TABLE 1:** Relative frequencies of acquired PAMs

PAM (5′–3′)	Frequency	%
TAAAT	35	21.2
TAAAA	23	13.9
CAAAT	19	11.5
AAAAT	18	10.9
CAAAA	11	6.7
TAAGT	10	6.1
AAAAA	7	4.2
TAAAG	7	4.2
AAAAG	3	1.8
AAAGT	3	1.8
TTAAA	3	1.8
TAAAC	2	1.2
GAAAT	2	1.2
AAAAC	1	0.6
AAATT	1	0.6
AAATC	1	0.6
AAACC	1	0.6
AAAGG	1	0.6
AAATG	1	0.6
AAGTG	1	0.6
AAGCT	1	0.6
ATAAA	1	0.6
TAATA	1	0.6
TAACA	1	0.6
TAAGA	1	0.6
TAAGC	1	0.6
TACAG	1	0.6
TCAAA	1	0.6
TCGCC	1	0.6
TGAAA	1	0.6
CAAAC	1	0.6
CAAAG	1	0.6
CAAGT	1	0.6
CAAGG	1	0.6
GAAAC	1	0.6

### Phage resistance assays.

As indicated above, between one and seven new spacers were acquired per BIM. It was previously shown that the acquisition of multiple spacers increases the overall phage resistance (26). Thus, we evaluated whether the number of acquired spacers influences the level of resistance toward phage M102AD. This was accomplished by comparing the phage titers on the various BIMs and the phage-sensitive wild-type (WT) host. No effect on the number of newly acquired spacers was observed, as all tested BIMs were fully phage resistant.

One striking observation was that one of the BIMs acquired a single spacer that did not target phage M102AD, yet the strain was still fully resistant to this phage. In this BIM, the phage resistance was non-CRISPR related, and another resistance mechanism is likely at play. Another frequent defense system leading to BIMs is the mutation of a phage receptor at the cell surface (12). To investigate this possibility, phage adsorption assays were performed using the WT strain and 11 BIMs that have acquired various spacers. While adsorption of phage M102AD to the WT strain was at 84% after 15 min of coincubation, adsorption to the BIMs was reduced between 5% and 57% (Table 2). The genome of one adsorption-resistant BIM was fully sequenced (BIM5). Two mutations were found in BIM5 compared to the WT sequence, outside the CRISPR locus. However, PCR amplifications of these regions in other BIMs did not show the same mutations.

**TABLE 2:** Phage adsorption assay

Strain	No. of spacers	Spacers acquired (5′–3′)	Targeted sequence on M102AD genome	% phage M102AD adsorption^*a*^
WT	NA^*b*^	NA	N/A	84 ± 1
BIM4	7	sp3, AACGCTCTGATTTCGTGTTTGTGTTATCGCC	14311–14284	54 ± 4
		sp4, GTCAAGCATACGTATATATGCTTGTGCACT	25547–25518	
		sp5, TGATGAAGTAAACCTCTTTTGTGAAAGGATT	24368–24338	
		sp6, TTAGCGCGAGTGATGATGGGTTGGTAATTGCC	16891–16922	
		sp7, CTCTAGCTTTATCTATTTTGATAAAGACAC	14862–14891	
		sp8, GTACACTCTGCAACTAACCCATCGGCACCA	7587–7616	
		sp9, CACGTCGAGTAAAATTGTACTAGCGCCTAA		
		sp10, AACGCTCTGATTTCGTGTTTGTGTTATCGCC	14314–14284	
		sp4, GTCAAGCATACGTATATATGCTTGTGCACT	25547–25518	
BIM5	7	sp5, TGATGAAGTAAACCTCTTTTGTGAAAGGATT	24368–24338	35 ± 5
		sp6, TTAGCGCGAGTGATGATGGGTTGGTAATTGCC	16891–16922	
		sp7, CTCTAGCTTTATCTATTTTGATAAAGACAC	14862–14891	
		sp8, GTACACTCTGCAACTAACCCATCGGCACCA	7587–7616	
		sp9, CACGTCGAGTAAAATTGTACTAGCGCCTAA		
BIM6	2	sp11, AAATTTTATAGCATATGCGAATATTGTTGT	27729–27700	56 ± 5
		sp12, GTTAACCGCAAGCGTAAAGTTTGCATATGC	27896–27867	
BIM7	4	sp13, AGAATTTTTCCATTCTTGCTCTTGGTTGGT	15737–15708	38 ± 3
		sp14, AGATGATAGTGACTTGTTTGCGGTAATTAA	3074–3103	
		sp15, AACTCTAACACTGGCTATTACTGATAAGAC	15938–15967	
		sp16, GAATTTTTCCATTCTTGCTCTTGGTTGGTT	15736–15707	
BIM9	3	sp18, TGGTTTGCACATTTTTTTTCCTTCCTTTTT	26698–26669	20 ± 7
		sp19, TAAGATTACATTTTGCAAGTAATCTTTCTT	22856–22827	
		sp21, GAATTGGGTTTTCCACAGTAGTAGCAAAGA	294–265	
BIM24	5	sp38, GCTAGTGACGTTAAAGATTTTGATGATAAT	25233–25262	57 ± 12
		sp39, TAAACACAAAGAAGCTTTGCAAGCCGTCGG	10138–10167	
		sp40, GCAGACAAAAGCTAAACAAGCTCTTGACTAT	8584–8614	
		sp41, GCTACTCGTATGTTGGATGTTATCGACGCC	9878–9907	
		sp1, CTCTTTTAGCAATTGTGAAAGGACGTAATT	24306–24335	
BIM29	3	sp11, AAATTTTATAGCATATGCGAATATTGTTGT	27729–27700	5 ± 3
		sp51, ACGATGAAGTAAACCTCTTTTGTGAAAGGATT	24367–24338	
		sp52, CTAAAACCGCAAGACACAGAGCCACAGGCT	4463–4492	
BIM31	3	sp54, TTCTTTACTAGTAAAACTTCTGTACATTTA	196–167	17 ± 17
		sp51, ACGATGAAGTAAACCTCTTTTGTGAAAGGATT	24367–24338	
		sp52, CTAAAACCGCAAGACACAGAGCCACAGGCT	4463–4492	
BIM33	2	sp56, GCCTTTAGACGAATGTATCCAAAATGTATCC	22183–22213	9 ± 2
		sp55, CATTAAAAGCTATGCGCAATAAGGACTATGT	26770–26799	
BIM34	6	sp57, GTTTTGGCTGTAACGTCTTTGACAACGCCG	5500–5471	9 ± 9
		sp58, TTTTGTAACTGCGTATCATCAGCGCTCGAG	16567–16538	
		sp51, ACGATGAAGTAAACCTCTTTTGTGAAAGGATT	24367–24338	
		sp59, CTTGCGATGTGGACAAATTGGGGCACGGTCA	6897–6927	
		sp51, ACGATGAAGTAAACCTCTTTTGTGAAAGGATT	24367–24338	
		sp52, CTAAAACCGCAAGACACAGAGCCACAGGCT	4463–4492	
BIM35	4	sp60, TAAAGCGTTTTGGATTAACTGCGCTTTAGC	4783–4754	10 ± 10
		sp61, CGCATAGAGTTTTGAGAGGTGAAGAATGTTT	23905–23935	
		sp51, ACGATGAAGTAAACCTCTTTTGTGAAAGGATT	24367–24338	
		sp52, CTAAAACCGCAAGACACAGAGCCACAGGCT	4463–4492	

a *n* = 2.

b NA, not applicable.

### Plasmid interference assays.

To confirm the CRISPR interference activity, we designed a plasmid-based assay. In the first experiment, the construct pNZ123-sp1 was transformed into S. mutans P42S WT. This construct harbors a protospacer sequence that is targeted by one of the native spacers already present in the CRISPR locus of the S. mutans P42S WT strain, along with the most common PAM sequence (TAAAT). Therefore, if the interference activity of the type II-A CRISPR-Cas system of S. mutans P42S is functional, the transformation of this plasmid should be prevented or greatly reduced. Indeed, while pNZ123 was transformable in this strain, pNZ123-sp1 was not transformable, validating the CRISPR interference activity as well as the PAM (Table 3).

**TABLE 3:** Plasmid interference assays^*a*^

Strain	Construct name	Insert sequence (5′–3′)	No. of clones per 10 μg plasmid DNA^*b*^
WT	pNZ123	NA	110 ± 28
	pNZ123-sp1	AAATATTTGAAAATTGTTTTTCACTAGATA-*TAAAT*	0
BIM1	pNZ123	NA	71 ± 30
	pNZ123-sp2	CTCTTTTAGCAATTGTGAAAGGACGTAATT-*TAAAT*	0
BIM2	pNZ123	NA	33.5 ± 7.5
	pNZ123-sp3	TTTTGGTCTAAAATTCTCAGGAATTTCACC-*TAAAT*	0

a No CSP was used during this experiment.

b *n* = 2.

A similar experiment was performed by transforming pNZ123 and the construct pNZ123-sp2 into BIM1. This S. mutans BIM derivative acquired a single spacer at the 5′ end of the CRISPR array from the phage M102AD genome, and the matching protospacer (and its PAM) was cloned into pNZ123-sp2. Transformation of pNZ123 was successful in BIM1 but transformation of pNZ123-sp2 was prevented, confirming the CRISPR-Cas interference activity and the PAM (Table 3).

To confirm that ectopically acquired spacers also conferred protection, we used pNZ123-sp3 and BIM2. This derivative strain acquired a single spacer between the native spacers 4 and 5 of the P42S CRISPR array. pNZ123-sp3 was constructed by cloning the matching protospacer and its PAM into pNZ123. Transformation of pNZ123 was successful in BIM2, but transformation of pNZ123-sp3 was inhibited, confirming that the ectopic spacer within the CRISPR array of S. mutans P42S provides protection against invading nucleic acids (Table 3).

### Plasmid-based determination of PAM sequence.

Because the above-mentioned plasmid interference assay was effective, we further investigated the 3′-end PAM associated with the type II-A CRISPR-Cas system of S. mutans P42S. Although there was evidence that the PAM is either NAA or NAAA, a plasmid interference assay was designed in which plasmid constructs with various PAMs were transformed. Each pNZ123-derived construct harbored a protospacer sequence targeted by the native spacer at the 5′ end of the CRISPR locus of S. mutans P42S. The protospacer was flanked by a 5-bp PAM, which was modified by at least 1 bp. A total of 18 constructs with alternative PAMs were transformed into P42S. Plasmids pNZ123 and pNZ123-sp1 were used as controls (Table 4).

**TABLE 4:**
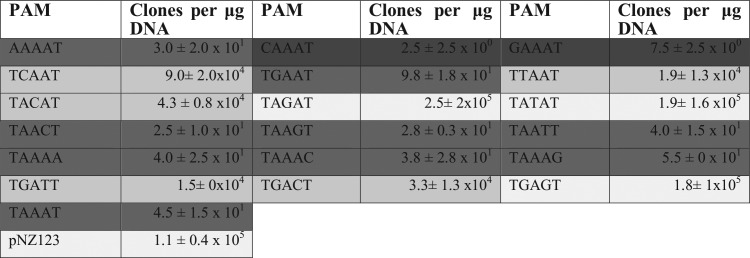
Plasmid interference assays to determine the PAM^*a*^

a The darker the boxes, the stronger the interference. CSP was used during this experiment. *, *n* = 2.

When the PAM was changed for any other base pair on position 1, 4, or 5, the interference activity was not affected and the transformation efficiencies of these plasmids were similar to that of pNZ123-sp1. This implies that these nucleotides do not play a significant role in CRISPR recognition. However, when the nucleotide was changed at positions 2 and 3, there was a drastic change in transformability. If any nucleotide other than the adenine was found at position 3, the CRISPR-Cas system did not interfere with transformation. If the adenine located at position 2 was exchanged for either a cytosine or a thymine, the same phenomenon occurred. However, if the adenine at position 2 was exchanged for a guanine, transformation interference was noted.

To investigate the importance of the flanking nucleotides when a guanine is at position 2 of the PAM, additional series of plasmid constructs were made in which the adenine at position 4 also was changed. When the transformation efficiencies of the new constructs were compared with the previously described plasmids, it became apparent that the guanine at position 2 was accepted only if the nucleotide at position 4 was an adenine.

## DISCUSSION

**FIG 4:**
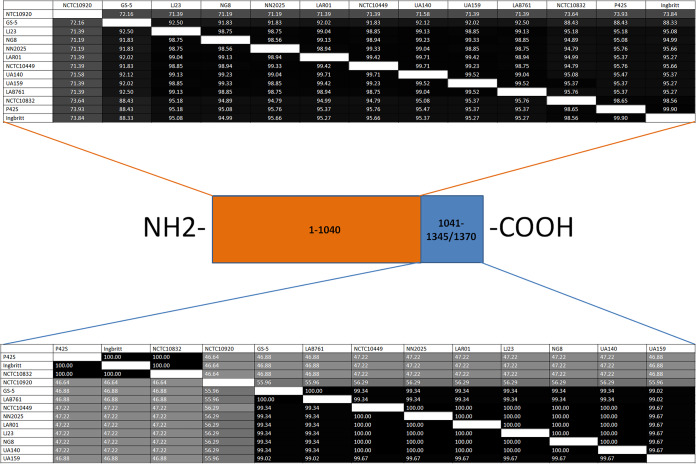
Percent identity between Cas9 N terminus and C terminus found in several S. mutans strains.

A functional type II-A CRISPR-Cas system was uncovered in the genome of S. mutans strain P42S. The *tracrRNA* as well as the *cas1*, *cas2*, and *csn2* genes shared a high percentage of identity with the similar loci found in other S. mutans genomes. Less conservation is found between the *cas9* genes. Functional and structural studies have previously shown that nuclease domains of Cas9 are found in the first part of the protein, which appears to be highly conserved in S. mutans. The C terminus of Cas9 is where the PAM-interacting domain is usually found (33, 34). Previously, the PAM recognized by the type II-A system of S. mutans strain UA159 was determined to be NGG based on DNA cleavage assays with purified Cas9 (35). While the C-terminal sequence of UA159 Cas9 has 99% or more identity with the same Cas9 region found in eight other S. mutans strains, the identity significantly drops compared to that of the Cas9 C terminus from strains P42S, Ingbritt, and NCTC10832 (46.9%) and from NCTC10920 (56.0%) (Fig. 4). These data suggest that the latter Cas9 proteins are structurally different in terms of their PAM recognition domain and, therefore, likely recognize different PAMs, which was experimentally confirmed here with Cas9 of strain P42S.

The adaptive nature of the CRISPR-Cas system of S. mutans P42S was also confirmed when spacer acquisition was observed after exposure to the virulent siphophage M102AD. Ectopic spacer acquisition was also noted, a phenomenon previously described in S. thermophilus. In a particular S. thermophilus strain in which BIM derivatives had acquired new ectopic spacers, the leader sequence had a deletion of the last base pair before the beginning of the first repeat compared to other strains (32). In comparisons of this last base pair of the leader sequence of BIMs of S. mutans P42S that have acquired ectopic spacers, no such deletion was observed. In S. thermophilus, the dinucleotide AG was at the 3′ end of the spacers found upstream of the newly acquired ectopic spacers (32). The same AG dinucleotide was found in spacer 4 of S. mutans P42S, which preceded most of the ectopic spacers that were acquired in the strain but not in spacer 1.

Phage adsorption assays revealed that in parallel to spacer acquisition, surface receptor mutations likely also play a role in phage resistance in S. mutans. This finding is in sharp contrast to those for S. thermophilus, where surface receptor mutations are less frequent (36, 37). The receptor for phage M102AD is currently not known, and no universal mutation responsible for adsorption resistance could be found. Of interest, in Streptococcus thermophilus it was already shown that multiple point mutations in the genome of various BIMs can result in phage resistance. One of these mutations was a specific point mutation in the methionine aminopeptidase gene, which also resulted in phage resistance in S. mutans P42S (37). Taken together, a variety of mutations is likely responsible for the phenotype.

The interference activity of the CRISPR-Cas system of S. mutans P42S was also confirmed using several plasmid constructs that harbored protospacers targeted by the native spacers. The data obtained from the spacer acquisition and interference assays led to the identification of a PAM at the 3′ end of the protospacer. First, we showed that 93% of the protospacers acquired from the genome of phage M102AD were flanked by 5′-NAA-3′, while 79% were flanked by 5′-NAAA-3′. The plasmid interference assays then revealed that the type II-A CRISPR-Cas system of S. mutans P42S interferes only when the targeted protospacer sequences are flanked at the 3′ end by NAA or NGAA. While the PAM sequence of NAA was identified in both spacer acquisition and interference activities, the NGAA sequence was only observed in one spacer acquisition event (spacer 145 in BIM93). Interestingly, the dinucleotide AA is found 6,828 times in the AT-rich genome of phage M102AD, while GAA is found 1,150 times. While there is a significant number of protospacers in the 30,664-bp genome of this phage, the PAM difference associated with the frequency of spacer acquisition is intriguing.

It was previously suggested for type I systems that PAM recognition during the interference stage occurs through a different mechanism than that during the acquisition stage, since the PAM requirements during the interference stage are less stringent (38, 39). However, in these systems, the expression of Cas1 and Cas2 is sufficient for spacer acquisition (40–43). In type II-A systems, Cas1 is not able to recognize PAM sequences to guide adaptation, while Cas1, Cas2, Csn2, and Cas9 all have been reported to be essential for spacer acquisition. The PAM recognition domain of Cas9 was shown to be involved during spacer acquisition but not its nuclease activity (44, 45). Whereas differences in PAM requirements in adaptation and interference may be explained by the different proteins involved in the two stages in type I systems, in type II-A systems PAM recognition occurs through Cas9 in both stages.

The finding of a type II-A CRISPR-Cas system using a distinct PAM may also have biotechnological applications. For example, one of the limitations of the current CRISPR-Cas9 genome editing technology, which mostly uses Cas9 of Streptococcus pyogenes, is the reliance on its NGG PAM, which limits the sequences that can be targeted (46). Cas9 of S. pyogenes (SpyCas9) has been engineered at the so-called PAM interacting motif to recognize other sequences, such as NAAG (47). Overall, amino acid sequence identity is very low between S. mutans Cas9 (SmCas9) and SpyCas9. When focusing on the PAM interacting motif of SpyCas9 and its NAAG-recognizing variant, no motif with any significant identity could be found with SmCas9. To date, type II-A Cas9 proteins naturally recognizing AT-rich PAMs, such as NAAAA, have been found in Lactobacillus buchneri (48) and Treponema denticola (49). The Cas9 protein of Streptococcus macacae (SmaCas9) has also been shown to recognize the shorter NAA PAM (50). The Cas9 of S. mutans P42S is 76.5% identical to SmaCas9 (75.3% N terminal, 80.9% C terminal).

An efficient Cas9 protein that can recognize the NAA or NGAA sequence would allow a wider range of targets for genome editing. For example, the human genome is known to be AT rich (51), and the Cas9 protein of S. mutans P42S may offer additional biotechnological benefits.

## MATERIALS AND METHODS

### Strain, phage, and culture conditions.

S. mutans strain P42S and the lytic phage M102AD were obtained from the Félix d’Hérelle Reference Center for Bacterial Viruses (www.phage.ulaval.ca). The bacterial strain was grown in brain heart infusion (BHI) medium at 37°C with 5% CO_2_. For growth on plates, 1.25% agar was added to BHI medium. Phage M102AD was amplified using an exponentially growing culture of P42S. Phage-infected cultures were incubated at 37°C until lysis. The resulting lysate was then filtered (0.45 μm) and stored at 4°C until use. Phage titration was performed using the double-layer plaque assay, and the top agar consisted of BHI medium supplemented with 0.75% agar.

### Identification and analysis of the CRISPR-Cas system in S. mutans P42S.

The *cas* genes and the CRISPR array sequences were obtained from the whole-genome sequence analysis of S. mutans P42S. The genomic DNA was first extracted as described elsewhere (52), with the following modifications. Briefly, the proteinase K and SDS steps were separated into a 15-min proteinase K (0.4 mg/ml) step, followed by a 2-h SDS (1%) step. After the potassium acetate step and subsequent centrifugation, the supernatant was treated with RNase A (2 μg/ml) for 1 h at 37°C. The protocol then was resumed with the isopropanol step as described previously (52). The genomic DNA of S. mutans P42S was prepared for sequencing using the Nextera XT DNA library preparation kit according to the manufacturer’s instructions. The library was sequenced on a MiSeq apparatus using a MiSeq reagent kit v2 (Illumina). Sequences were assembled into 18 contigs using Ray Assembler 2.3.0 (53) and fused using Mauve Assembly Metrics (54). CRISPR loci were identified by searching for repeat sequences as listed in the CRISPR database (https://crispr.i2bc.paris-saclay.fr/). The *cas* gene sequences from other S. mutans strains were obtained from NCBI and compared to the genome of S. mutans P42S. Clustal Omega (http://www.ebi.ac.uk/Tools/msa/clustalo/) was used to determine the percent identity between the DNA sequences and between the translated amino acid sequences.

### BIM assay.

An overnight culture of S. mutans P42S was transferred (1%) to fresh BHI medium and grown to an optical density at 600 nm (OD_600_) of 0.3 to 0.5. Mixtures of 100 μl of the S. mutans P42S culture and 100 μl of phage M102AD lysate (titer between 10^7^ and 10^9^ PFU/ml) then were mixed in BHI top agar and poured directly onto solid medium. Plates were incubated at 37°C for 48 to 72 h, and surviving cells were analyzed for spacer acquisition by amplifying the CRISPR locus as described elsewhere (16). The primers CR-F (5′-AATGTCGTGACGAAAATTGG-3′) and CR-R (5′-GAAGTCATCGGAACGGTCAT-3′) were used to amplify the CRISPR locus found in S. mutans P42S. PCR products were sequenced with an ABI 3730xl analyzer at the Plateforme de Séquençage et de Génotypage des Génomes at the CHU of Québec City. BIM assays were also performed with UV-damaged phage lysates as described previously (19).

### Phage adsorption assay.

An overnight culture of S. mutans P42S was transferred (1%) to fresh BHI medium and grown until an OD_600_ of 0.7. Phage M102AD (10^3^ PFU) was added to 900 μl of this culture and allowed to adsorb for 15 min at 37°C. Cultures then were centrifuged for 1 min at maximum speed in a microcentrifuge, and the titer of the supernatant was determined to estimate the phage fraction that did not adsorb to the host cells.

### Plasmid interference assay.

An approach similar to the one described by Serbanescu et al. was performed (30), except that we used plasmid pNZ123 (55). pNZ123 contains 2,497 bp, provides chloramphenicol resistance to the host cells, and is readily transformable in S. mutans. The 24 bp between the XhoI and EcoRI restriction sites (positions 149 to 173) were removed, and the resulting linearized plasmid (2,473 bp) was purified from an agarose gel using the QIAquick gel purification kit as described by the manufacturer. Various DNA inserts were ligated between the XhoI and EcoRI sites of the gel-purified plasmid (see below).

A 30-bp protospacer sequence, targeted by one of the spacers already present in the CRISPR locus of the wild-type (WT) strain S. mutans P42S and flanked by the nucleotide sequence TAAAT (see below) at the 3′ end, was first cloned between the XhoI and EcoRI sites to generate pNZ123-sp1. The recombinant plasmid was confirmed by sequencing. pNZ123 and pNZ123-sp1 then were independently transformed (see below) into S. mutans P42S.

Another 30-bp protospacer targeted in strain BIM1 was cloned between the XhoI and EcoRI sites of pNZ123. The cloned protospacer was flanked by 5 bp found downstream in the phage genome (positions 24306 to 24335) to generate pNZ123-sp2. pNZ123 and pNZ123-sp2 were independently transformed into S. mutans P42S BIM1.

Finally, the activity of ectopically acquired spacers (see below) was assayed by a similar experiment. The 30-bp protospacer (positions 18743 to 18714) targeted in BIM2 and flanked by the 5 bp downstream was cloned between the XhoI and EcoRI sites of pNZ123 to generate pNZ123-sp3. pNZ123 and pNZ123-sp3 then were independently transformed into S. mutans P42S BIM2.

### Transformation of S. mutans.

The plasmid constructs were transformed into S. mutans using natural competence (56) through the addition of the competence-stimulating peptide (CSP). The active form of this peptide has the following sequence: NH_2_-SGSLSTFFRLFNRSFTQA-COOH (57, 58). The first peptide batch was kindly provided by Céline Lévesque from the University of Toronto. All subsequent batches were ordered from Biomatik (www.biomatik.com). An overnight culture of S. mutans P42S was transferred to fresh BHI medium and grown at 37°C until the OD_600_ reached 0.1. Aliquots of 500 μl then were collected and 1 μg of plasmid DNA was added to them. Along with the plasmid construct, the CSP was added at a concentration of 1 μM to the growing culture (59). If no CSP was added, the quantity of plasmid DNA was increased to 10 μg. The cultures were incubated at 37°C and 5% CO_2_ for 2.5 h and spun down, and the cell pellets were resuspended in 100 μl of BHI. Samples were plated onto BHI agar plates supplemented with 10 μg/ml chloramphenicol. Plates were incubated at 37°C for 72 h.

### Determination of PAM sequence.

Based on the CRISPR analysis of various S. mutans BIMs obtained after the challenge with the virulent phage M102AD, we identified several newly acquired spacers. The analysis of the sequences flanking the protospacers (26) in the genome of phage M102AD led to the identification of a preferred 5-bp PAM motif at the 3′ end of the protospacer. To determine the importance of each of these five base pairs, we designed a plasmid-based interference experiment as described above. Between the XhoI and EcoRI sites of pNZ123, one of the spacers already present in the CRISPR locus of S. mutans P42S was flanked by a 5-bp motif and several derivatives. The pNZ123-sp1 plasmid was used as a control, as it contains the protospacer flanked by the most commonly observed PAM (TAAAT). Other versions of the plasmid included one or two mismatches in the motif, as a nucleotide was replaced by one of the three other alternatives. All plasmids were transformed in duplicate into S. mutans P42S WT and derivatives, and their transformability was compared. The constructs and insert sequences are listed in Table S2 in the supplemental material.

10.1128/mSphere.00235-20.2 TABLE S2 Construct insert sequences. Protospacer sequences with flanking PAM are in italics. Download Table S2, DOCX file, 0.02 MB. Copyright © 2020 Mosterd and Moineau. 2020 Mosterd and Moineau This content is distributed under the terms of the Creative Commons Attribution 4.0 International license.

### Data availability.

The complete genome sequence of phage M102AD was previously deposited in GenBank under accession number DQ386162 (10). The sequences of the *cas* genes of S. mutans P42S are available in GenBank under accession numbers MT008463 (*cas9*), MT008464 (*cas1*), MT008465 (*cas2*), MT008466 (*csn2*), and MT008467 (*tracrRNA*).
