# Supplementary figures and images for: Characterization of a Type II-A CRISPR-Cas System in Streptococcus mutans
Source: mSphere. 2020 Jun 24;5(3):e00235-20. doi: 10.1128/mSphere.00235-20 (PMC7316486; doi:10.1128/mSphere.00235-20)

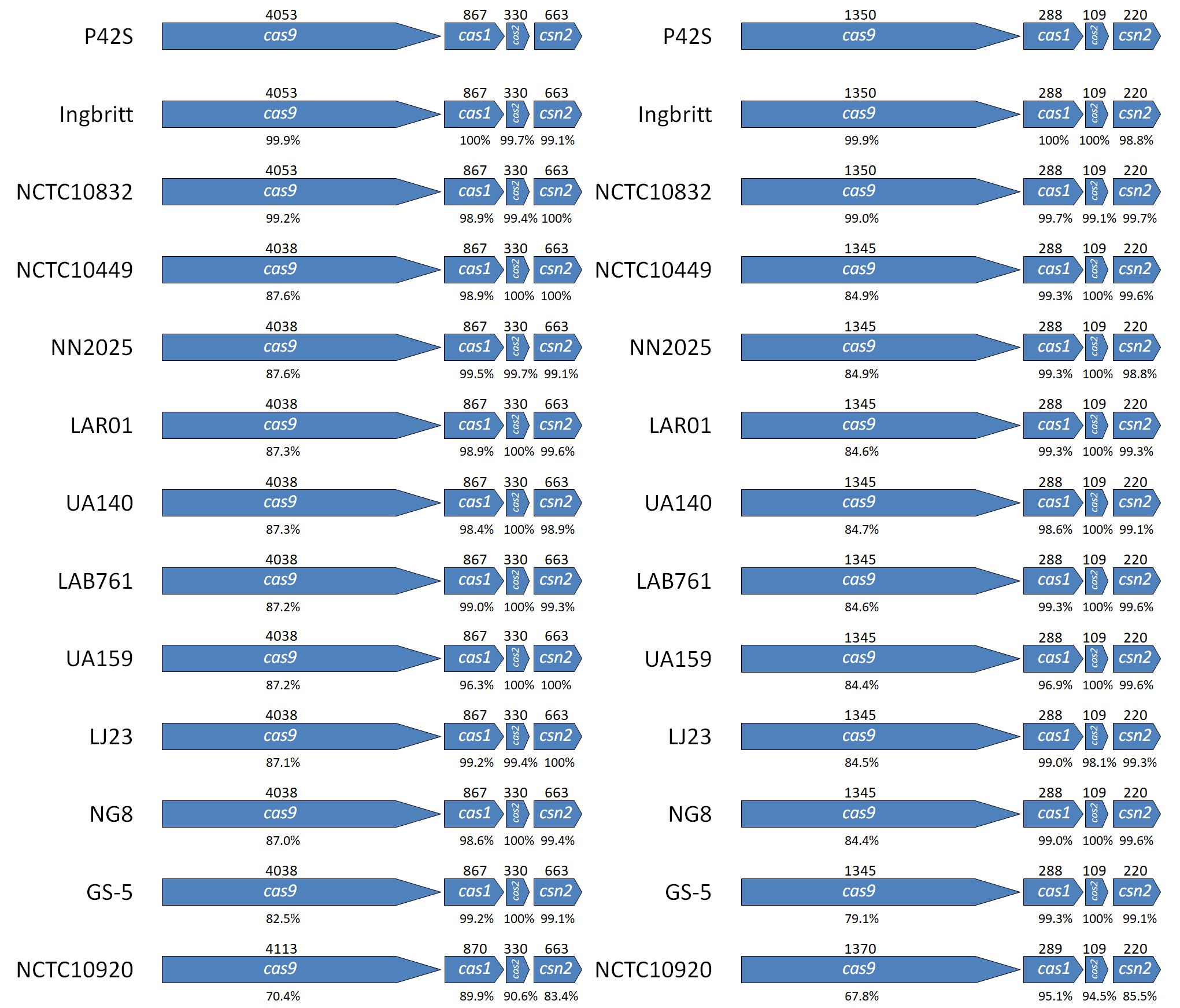

Supplement: FIG S1 [file mSphere.00235-20-sf001.tif]
