# Supplementary material for: Characterization of a Type II-A CRISPR-Cas System in Streptococcus mutans
Source: mSphere. 2020 Jun 24;5(3):e00235-20. doi: 10.1128/mSphere.00235-20 (PMC7316486; doi:10.1128/mSphere.00235-20)
Supplement: TABLE S1 [file mSphere.00235-20-st001.docx]

| **Spacer** | **Position in phage genome (5’-3’)** | **Size (nt)** | **Times acquired** | **Proto-spacer sequence (5’-3’)** | **PAM** |
| --- | --- | --- | --- | --- | --- |
| 1 | 24306-24335 | 30 | 19 | CTCTTTTAGCAATTGTGAAAGGACGTAATT | TAAAT |
| 2 | 18743-18714 | 30 | 1 | TTTTGGTCTAAAATTCTCAGGAATTTCACC | TAAAT |
| 3 | 14311-14284 | 28 | 1 | GCTCTGATTTCGTGTTTGTGTTATCGCC | TAAGT |
| 4 | 25547-25518 | 30 | 4 | GTCAAGCATACGTATATATGCTTGTGCACT | CAAAT |
| 5 | 24368-24338 | 31 | 4 | TGATGAAGTAAACCTCTTTTGTGAAAGGATT | TAAAT |
| 6 | 16891-16922 | 32 | 4 | TTAGCGCGAGTGATGATGGGTTGGTAATTGCC | AAAAA |
| 7 | 14862-14891 | 30 | 4 | CTCTAGCTTTATCTATTTTGATAAAGACAC | AAAAA |
| 8 | 7587-7616 | 30 | 5 | GTACACTCTGCAACTAACCCATCGGCACCA | CAAAT |
| 9* | X | 30 | 4 |  |  |
| 10 | 14314-14284 | 31 | 2 | AACGCTCTGATTTCGTGTTTGTGTTATCGCC | TAAGT |
| 11 | 27729-27700 | 30 | 5 | AAATTTTATAGCATATGCGAATATTGTTGT | TAAAA |
| 12 | 27896-27867 | 30 | 1 | GTTAACCGCAAGCGTAAAGTTTGCATATGC | TAAGT |
| 13 | 15737-15708 | 30 | 1 | AGAATTTTTCCATTCTTGCTCTTGGTTGGT | TAAAT |
| 14 | 3074-3103 | 30 | 1 | AGATGATAGTGACTTGTTTGCGGTAATTAA | CAAAT |
| 15 | 15938-15967 | 30 | 1 | AACTCTAACACTGGCTATTACTGATAAGAC | TAAAA |
| 16 | 15736-15707 | 30 | 1 | GAATTTTTCCATTCTTGCTCTTGGTTGGTT | AAATC |
| 17 | 24343-24314 | 30 | 1 | AGGATTTAAATTACGTCCTTTCACAATTGC | TAAAA |
| 18 | 26698-26669 | 30 | 3 | TGGTTTGCACATTTTTTTTCCTTCCTTTTT | TAAAT |
| 19 | 22856-22827 | 30 | 4 | TAAGATTACATTTTGCAAGTAATCTTTCTT | TAAAT |
| 20 | 24402-24431 | 30 | 1 | GCAAAGAAGCGATTATGAAGCGTGCAGAAG | AAAAT |
| 21 | 294-265 | 30 | 1 | GAATTGGGTTTTCCACAGTAGTAGCAAAGA | TAAAG |
| 22 | 7700-7671 | 30 | 1 | GTATGCGCCTTCTGCATTTTCGACATATCC | TAAAA |
| 23 | 23484-23513 | 30 | 1 | CTGTACAGTAGGATTTTTAAAGGCTCTACT | AAAAA |
| 24* | 23555-23584 | 31 | 1 | GATAAAATTTTAATGCGACAAACAGAAATC | AAAAA |
| 25 | 8860-8889 | 30 | 1 | CAATCAAACAGCAGAAAGCCTTGCTAAAGC | AAAAT |
| 26 | 12350-12320 | 31 | 1 | CAAGATGGCTGAAGAGACCGGGGTTAACGCC | TAAAT |
| 27 | 27519-27490 | 30 | 1 | TAACGCGTTCATAATGTTGAGCAAGCCAGC | TAAAT |
| 28 | 4067-4097 | 31 | 1 | TTTGCGGGCGCCGAGATATACAGCGAACTTA | AAGCT |
| 29 | 15738-15709 | 30 | 1 | CAGAATTTTTCCATTCTTGCTCTTGGTTGG | TTAAA |
| 30* | X | 30 | 2 |  |  |
| 31 | 19453-19424 | 30 | 2 | ATACTAATAATTTCAGGCTTTAACAAATCA | TAAAT |
| 32 | 15108-15078 | 31 | 3 | TAGTAAAGTCAAAAGTAACTGCATTAGTCTT | TAAAT |
| 33 | 24486-24456 | 31 | 1 | CCTTCTTTAGTTTCAACTACAATTCCAGCCT | CAAAT |
| 34 | 15510-15481 | 30 | 1 | CTAGCGTATTGTCTTTATCTGTTTTTAACT | CAAAT |
| 35 | 10673-10644 | 30 | 2 | CTGTAATTAGACCGCCCAAGCCATGCTTGT | TAAAT |
| 36 | 24485-24456 | 30 | 1 | CTTCTTTAGTTTCAACTACAATTCCAGCCT | CAAAT |
| 37 | 23729-23759 | 31 | 1 | GAAGAATTGCAAGCAAAAATTGAAGAGGTGT | TAAAT |
| 38 | 25233-25262 | 30 | 1 | GCTAGTGACGTTAAAGATTTTGATGATAAT | CAAAA |
| 39 | 10138-10167 | 30 | 1 | TAAACACAAAGAAGCTTTGCAAGCCGTCGG | CAAGG |
| 40 | 8584-8614 | 31 | 1 | GCAGACAAAAGCTAAACAAGCTCTTGACTAT | CAAAA |
| 41 | 9878-9907 | 30 | 1 | GCTACTCGTATGTTGGATGTTATCGACGCC | AAAAT |
| 42 | 5035-5065 | 31 | 1 | TGAACGATTTGGGAAAAATAAAAAAAGCTCT | TAAAA |
| 43 | 26558-26587 | 30 | 5 | ACTTAACTCATTATAAAAAGCGTTACAGAG | AAAAT |

| 44 | 27509-27538 | 30 | 1 | TGAACGCGTTATATATACGTATAACGTTAA | GAAAT |
| --- | --- | --- | --- | --- | --- |
| 45 | 680-709 | 30 | 1 | GTAATGGCGGTTGAAAATATTCGCAGAGCT | TACAG |
| 46 | 15454-15483 | 30 | 1 | TTGCAAGTCAATTTGATTGTGAATTTGAGT | TAAAA |
| 47* | 23269-23240 | 31 | 1 | TCGACTACATTCACAAACGCCGCTGTCTTA | TCGCC |
| 48 | 21368-21339 | 30 | 1 | ACATAGCCAGTGTGACCGAAAGGGTGGCCG | TAAAG |
| 49* | 24306-24335 | 31 | 1 | CTCTTTTAGCAATTGTGAAAGGACGTAATT | TAAAT |
| 50 | 24040-24011 | 30 | 1 | GCCAACGCACTCATGTCTTTGTGCCGTTAA | AAAAT |
| 51* | 24367-24338 | 32 | 5 | GATGAAGTAAACCTCTTTTGTGAAAGGATT | TAAAT |
| 52 | 4463-4492 | 30 | 5 | CTAAAACCGCAAGACACAGAGCCACAGGCT | CAAAA |
| 53 | 21422-21452 | 31 | 1 | ATGACAGCAATTTTTACACTGGCACAGTAGC | TAAAT |
| 54 | 196-167 | 30 | 2 | TTCTTTACTAGTAAAACTTCTGTACATTTA | TAAAA |
| 55* | 26770-26799 | 31 | 2 | ATTAAAAGCTATGCGCAATAAGGACTATGT | TAAAT |
| 56 | 22183-22213 | 31 | 1 | GCCTTTAGACGAATGTATCCAAAATGTATCC | AAAGT |
| 57 | 5500-5471 | 30 | 1 | GTTTTGGCTGTAACGTCTTTGACAACGCCG | TAAAA |
| 58 | 16567-16538 | 30 | 1 | TTTTGTAACTGCGTATCATCAGCGCTCGAG | TAAGT |
| 59 | 6897-6927 | 31 | 1 | CTTGCGATGTGGACAAATTGGGGCACGGTCA | AAATG |
| 60 | 4783-4754 | 30 | 1 | TAAAGCGTTTTGGATTAACTGCGCTTTAGC | AAAAA |
| 61 | 23905-23935 | 31 | 1 | CGCATAGAGTTTTGAGAGGTGAAGAATGTTT | AAAAT |
| 62 | 27259-27288 | 30 | 1 | GCAGAAAAAGAAGAACTGATAAGCGATATT | AAAAA |
| 63 | 24244-24274 | 31 | 1 | TGGAAATACTTTGCGAGATTTTGTAGACCCG | CAAAA |
| 64* | 7587-7616 | 31 | 1 | GTACACTCTGCAACTAACCCATCGGCACCA | CAAAT |
| 65 | 28219-28190 | 30 | 1 | ATTTTAAACATATTTTCTCACCACCTTTTT | TAAAA |
| 66 | 27520-27490 | 31 | 2 | ATAACGCGTTCATAATGTTGAGCAAGCCAGC | TAAAT |
| 67 | 26598-26627 | 30 | 1 | AGTGAGAGTAGCTACAGTTGCAAGAGCTGA | TAAAT |
| 68 | 26670-26700 | 31 | 1 | AAAAGGAAGGAAAAAAAATGTGCAAACCAAT | CAAAA |
| 69 | 25601-25572 | 30 | 1 | CTCTAAGTCATAGTCTGCTTTAATTTGCAT | TAAAA |
| 70 | 6185-6214 | 30 | 2 | AGCTGCTACTATCTATGAGTACAGACAAGC | AAAAT |
| 71 | 6654-6625 | 30 | 1 | TATTTTCAGCCATGTTTTTCGGTTTCCTTA | CAAAT |
| 72 | 13969-13998 | 30 | 1 | GCGCCAAAAGCAAAAACGGAGCCAGTAATC | AAAAT |
| 73 | 10069-10098 | 30 | 1 | CGTTTTCAAGGCCTTCAGTGGTGACAAGAG | TAAAA |
| 74 | 29484-29513 | 30 | 1 | GATGACATCGCATTAATTACAATCAGTTTA | TAAGT |
| 75 | 10674-10645 | 30 | 1 | CCTGTAATTAGACCGCCCAAGCCATGCTTG | TTAAA |
| 76 | 19742-19713 | 30 | 1 | TGTCTGTTGCGTTAGTTATTAAGCTATCAA | TAAGT |
| 77 | 21056-21027 | 30 | 1 | GATACAAACAATAAACTAGCTGACAAACCT | AAAAT |
| 78 | 23097-23067 | 31 | 1 | TTTGCGCGTGCGTTTTAGCTTCAAAGTTATC | AAAAC |
| 79 | 9369-9340 | 30 | 3 | GCTTCGTCAGTTTGCCCAAAAGCATCTTGC | AAAGT |
| 80 | 4742-4771 | 30 | 1 | GATAATGTTTTTGCTAAAGCGCAGTTAATC | CAAAA |
| 81 | 9113-9084 | 30 | 1 | TGATTTTGCTAAGTGCACCACGAACAAGAT | TAAGC |
| 82 | 10549-10578 | 30 | 2 | CAAAACACTACAAGTTGCTTTTGCCCCCCT | CAAAT |
| 83* | 20065-20095 | 31 | 1 | AAGCCA CAGAAACAACTAC GCACCAGCA | GAAAC |
| 84 | 5182-5211 | 30 | 1 | ATACGAAACTTTCGAAATTAATGACAGTTG | TAAAT |
| 85 | 21258-21287 | 30 | 1 | ACGTGCAGGCTATGAGATTGTGCCAGCTAA | TAAAC |
| 86* | 24044-24015 | 31 | 1 | GTGCGCCAACGCACTCATGTCTTTGTGCCG | TTAAA |
| 87 | 29686-29716 | 31 | 1 | TCATTATGTTTACACAATATTACATTAGTAT | TAAGT |
| 88 | 18567-18538 | 30 | 1 | CCATATCTAGAATATTTTTCCCGTTGAGGT | AAAAG |
| 89 | 7577-7548 | 30 | 1 | GTTACCGTCGATACGTTCGATGGAACCTTC | TAAGT |
| 90 | 28701-28730 | 30 | 1 | GAAATTAGAAGCAGAAGAGATTTTCTGTGA | TAAAA |
| 91 | 27384-27355 | 30 | 2 | ATAAGTTTGAGCGACTTTTTTCGAAACCTT | TAAAT |
| 92 | 26049-26020 | 30 | 1 | CTCTGCGCGCTACTATCGCAATATCATGAT | TAAAA |
| 93 | 30543-30514 | 30 | 1 | AAAAAAATCACATAAAGGTAGTATTGTAGA | AAAAT |
| 94 | 23836-23807 | 30 | 1 | GCATAGCTTTTGTGCTATCACTGATTTTCC | AAAAG |

| 95 | 9783-9753 | 31 | 1 | CGCTTAAAACGCGCTTCTATCAGCGGGTCCG | AAACC |
| --- | --- | --- | --- | --- | --- |
| 96 | 17625-17596 | 30 | 1 | CGTTGCCAAAACTATATCTGATATCATGCC | TAAAA |
| 97 | 28220-28249 | 30 | 1 | CGAAAAATATAGACCTTTCCGAATTAATTA | CAAAT |
| 98 | 13968-13998 | 31 | 1 | AGCGCCAAAAGCAAAAACGGAGCCAGTAATC | AAAAT |
| 99 | 22318-22348 | 31 | 1 | GGAAAAGCAATTGTTAAGCGTCGAAGGAATT | AAAGG |
| 100 | 23531-23501 | 31 | 1 | TTTGTACACATCATTTTTAGTAGAGCCTTTA | AAAAT |
| 101 | 27730-27700 | 31 | 1 | AAAATTTTATAGCATATGCGAATATTGTTGT | TAAAA |
| 102 | 14469-14441 | 29 | 1 | CTTTGAACCAATGAACTTGTAAGGAGTCA | CAAAG |
| 103 | 15498-15469 | 30 | 1 | CTTTATCTGTTTTTAACTCAAATTCACAAT | CAAAT |
| 104 | 30423-30452 | 30 | 1 | ATGTCACATTATTAGAATACTAGAATATTC | TAATA |
| 105 | 18539-18570 | 32 | 1 | CCTCAACGGGAAAAATATTCTAGATATGGTAA | AAGTG |
| 106 | 5019-4989 | 31 | 1 | TAGCTTCTGCCGGGTCTGAAGTAGATAGCCC | AAAAT |
| 107 | 27731-27760 | 30 | 1 | CAGACGGATACCTGGCGTTATGTTATGCAG | CAAAT |
| 108 | 100-71 | 30 | 1 | ATTTCGCTTAATCCGATTGAATATCCGGTT | TAAAT |
| 109 | 19150-19121 | 30 | 1 | GGTTAATCCGCTGACTTGAATAGTTGCACT | CAAAT |
| 110 | 22555-22584 | 30 | 1 | GTATTTAAGACCAGAAACACTTTTCAGTAA | TAAAT |
| 111* | 9430-9459 | 32 | 1 | CAGACATGTTGTCCATCATCAATACATTTCC | TAAAA |
| 112 | 3522-3492 | 31 | 1 | TGGCAAATGTATATAGTTTTCGTTTAAGTTC | TAAAT |
| 113 | 21490-21461 | 30 | 1 | AACCTAACATGTAATCGCGTGTGCGGTGCA | TAAAT |
| 114* | 29177-29148 | 31 | 1 | AACCATCTGTTAGTTTTCTCATTAAATTT | CAAAA |
| 115 | 24367-24338 | 30 | 7 | GATGAAGTAAACCTCTTTTGTGAAAGGATT | TAAAT |
| 116 | 9920-9949 | 30 | 1 | GTGGCAAAAGTGATAGCTAGGAACATGCCT | CAAAT |
| 117 | 12260-12231 | 30 | 1 | GGTCAGCTCCTGCGTCTTTAGCTTTGTCTT | TAAAG |
| 118 | 1220-1249 | 30 | 1 | AGATAAGAAAGACGATAATCTAAGATTTAA | TAAAA |
| 119 | 29184-29155 | 30 | 1 | TTTCGAAAAACCATCTGTTAGTTTTCTCAT | TAAAT |
| 120* | 30034-30004 | 32 | 1 | CTGCGCTTCTCGAGTTTTATCGATAGACTCA | AAAAT |
| 121 | 27692-27721 | 30 | 2 | TTGTTTTAACAACAATATTCGCATATGCTA | TAAAA |
| 122 | 19103-19132 | 30 | 1 | CGTAGCTTCAACTATTTGAGTGCAACTATT | CAAGT |
| 123 | 17928-17899 | 30 | 1 | CGGCAGTTTTAGCACTTGCGGTAACTTGAC | TAAAG |
| 124 | 12349-12320 | 30 | 2 | AAGATGGCTGAAGAGACCGGGGTTAACGCC | TAAAT |
| 125 | 5097-5068 | 30 | 1 | CAATGTCGTTTTGCACAAAGTTTTGAGTTT | TAAGA |
| 126 | 29057-29086 | 30 | 1 | ACGCTTGATGCGCGCTTGGTTGTTTGATTA | CAAAA |
| 127 | 14150-14179 | 30 | 1 | CAGCTAAAAATGTTGCAATCAACACGCTTC | CAAAT |
| 128 | 13903-13932 | 30 | 1 | GGTCACGACAGCGATTATGGAACCATCACC | AAAAA |
| 129 | 24102-24132 | 31 | 1 | CCGAGTCTAGATTAACTATCAGTGTAGCGCT | AAAGT |
| 130 | 4611-4641 | 31 | 1 | ACAACGACGACGAGCAAGACGAAAAGCCTGA | TAAAG |
| 131* | X | 30 | 2 |  |  |
| 132 | 24305-24335 | 31 | 1 | GCTCTTTTAGCAATTGTGAAAGGACGTAATT | TAAAT |
| 133 | 5165-5195 | 31 | 1 | TGAACGTGTGCGTGACTATACGAAACTTTCG | AAATT |
| 134 | 20127-20156 | 30 | 1 | GCGCAGAGGTACCATCTGCAAACGAGATTT | TAAAA |
| 135 | 22694-22723 | 30 | 1 | GTGGCCGTTAAAAAGCAAGGAAGAATACGT | TAAAT |
| 136 | 26541-26570 | 30 | 2 | GCTAGCACGTTGAAAACACTTAACTCATTA | TAAAA |
| 137 | 21423-21452 | 30 | 1 | TGACAGCAATTTTTACACTGGCACAGTAGC | TAAAT |
| 138 | 29338-29367 | 30 | 1 | CAAAGTCTTTAAGCACAAACGAGCGACTGA | CAAAT |
| 139 | 26203-26173 | 31 | 1 | CAAAATAAAAAAGCTTATAGAAATAAGCTTT | AAAAT |
| 140 | 4074-4045 | 30 | 1 | CCCGCAAAATAAGAGCCACCGCCGGAGTTC | AAAAT |
| 141 | 19201-19230 | 30 | 1 | AAACAAGAAAATCTTTGTTCAGCTACTTCC | AAAAT |
| 142* | 27729-27700 | 31 | 1 | AAATTTTATAGCATATGCGAATATTGTTGT | TAAAA |
| 143 | 9523-9552 | 30 | 1 | TGAAGAGTATACAAAAATGCAGTCGGAAGG | CAAAA |
| 144 | 10070-10099 | 30 | 1 | GTTTTCAAGGCCTTCAGTGGTGACAAGAGT | AAAAT |
| 145* | 20010-20039 | 30 | 1 | TTAAAGACGAAAATTCACGCGG ACTGTAG | TGAAA |

| 146 | 6754-6725 | 30 | 1 | GCGAGCTCAGCAAACTTCTTTGCGCCTGCG | TAAGT |
| --- | --- | --- | --- | --- | --- |
| 147 | 25859-25888 | 30 | 1 | TGAAAGAAAGGTTAAGCTAATGCTAAAACT | AAAAG |
| 148 | 25628-25599 | 30 | 5 | TTTTTCTTTTTTCACTTTCTGCGCTTTCTC | TAAGT |
| 149* | 24367-24338 | 31 | 3 | GATGAAGTAAACCTCTTTTGTGAAAGGATT | TAAAT |
| 150 | 26090-26120 | 31 | 3 | ACTGGAAAACGGGCTCAAGGAGTTTAAACAG | CAAAT |
| 151 | 10581-10611 | 31 | 2 | AATTAATTGGCACTTTAGTTGTCCTACCTTT | TAAAA |
| 152 | 26863-26892 | 30 | 1 | TATACGTTATTTCAACTGGAAAATTTGCAA | CAAAT |
| 153 | 18921-18892 | 30 | 1 | CCAATTTCTAACATATTCTCACCCCCTTTC | GAAAT |
| 154 | 26142-26171 | 30 | 1 | CGACTGAGAAAATTGATGTCAATAGAATTT | TAAAG |
| 155* | 9920-9949 | 31 | 1 | GTGGCAAAAGTGATAGCTAGGAACATGCCT | CAAAT |
| 156 | 10923-10952 | 30 | 1 | AACAAACACAAAAAGACATACAGAAGATGA | TAAAA |
| 157* | 19006-18977 | 30 | 1 | TCCACTAATTT GTCATCACTAAAATCAAC | TAAAG |
| 158 | 3061-3031 | 31 | 1 | CGTGTCGTCAATGCAAATGTCGACGTAAAAT | CAAAC |
| 159 | 19971-20000 | 30 | 1 | AACAAATTAGTGACTTAATTGAGTCTGCCG | TAAAT |
| 160 | 29822-29793 | 30 | 1 | CAAAGCCTTTAGGCTTTGATATTTACTTGC | TAAAT |
| 161 | 9430-9459 | 30 | 1 | AGACATGTTGTCCATCATCAATACATTTCC | TAAAA |
| 162 | 1296-1325 | 30 | 1 | GCTAGCGTGCAGATTGATTATGATTTTCTT | ATAAA |
| 163* | 5314-5343 | 31 | 1 | TTGACGACTTAATCCAACTCGACTTTAACA | TCAAA |
| 164 | 21281-20310 | 30 | 1 | AATCAAACTGACTGAGTCGACTGGTTATTA | TAACA |
| 165 | 7685-7714 | 30 | 1 | TGCAGAAGGCGCATACGAACTTCAAGAGGT | TAAAC |
| 166 | 14911-14882 | 30 | 1 | AACTCAAATCTAATGTTTTTGTGTCTTTAT | CAAAA |
| 167 | 26285-26315 | 31 | 1 | GGTAAAAACCATGAAAACAATCGCAAAATTA | CAAAA |
| 168 | 24307-24335 | 29 | 1 | TCTTTTAGCAATTGTGAAAGGACGTAATT | TAAAT |

5
