# Supplementary material for: Characterization of a Type II-A CRISPR-Cas System in Streptococcus mutans
Source: mSphere. 2020 Jun 24;5(3):e00235-20. doi: 10.1128/mSphere.00235-20 (PMC7316486; doi:10.1128/mSphere.00235-20)
Supplement: TABLE S2 [file mSphere.00235-20-st002.docx]

| **Construct name** | **Insert sequence (5’-3’)** |
| --- | --- |
| pNZ123-sp1 | AAATATTTGAAAATTGTTTTTCACTAGATA-*TAAAT* |
| pNZ123-sp2 | CTCTTTTAGCAATTGTGAAAGGACGTAATT-*TAAAT* |
| pNZ123-sp3 | TTTTGGTCTAAAATTCTCAGGAATTTCACC-*TAAAT* |
| PAM1TA | AAATATTTGAAAATTGTTTTTCACTAGATA-*AAAAT* |
| PAM1TC | AAATATTTGAAAATTGTTTTTCACTAGATA-*CAAAT* |
| PAM1TG | AAATATTTGAAAATTGTTTTTCACTAGATA-*GAAAT* |
| PAM2AC | AAATATTTGAAAATTGTTTTTCACTAGATA-*TCAAT* |
| PAM2AG | AAATATTTGAAAATTGTTTTTCACTAGATA-*TGAAT* |
| PAM2AT | AAATATTTGAAAATTGTTTTTCACTAGATA-*TTAAT* |
| PAM3AC | AAATATTTGAAAATTGTTTTTCACTAGATA-*TACAT* |
| PAM3AG | AAATATTTGAAAATTGTTTTTCACTAGATA-*TAGAT* |
| PAM3AT | AAATATTTGAAAATTGTTTTTCACTAGATA-*TATAT* |
| PAM4AC | AAATATTTGAAAATTGTTTTTCACTAGATA-*TAACT* |
| PAM4AG | AAATATTTGAAAATTGTTTTTCACTAGATA-*TAAGT* |
| PAM4AT | AAATATTTGAAAATTGTTTTTCACTAGATA-*TAATT* |
| PAM5TA | AAATATTTGAAAATTGTTTTTCACTAGATA-*TAAAA* |
| PAM5TC | AAATATTTGAAAATTGTTTTTCACTAGATA-*TAAAC* |
| PAM5TG | AAATATTTGAAAATTGTTTTTCACTAGATA-*TAAAG* |
| PAM2AG4AT | AAATATTTGAAAATTGTTTTTCACTAGATA-*TGATT* |
| PAM2AG4AC | AAATATTTGAAAATTGTTTTTCACTAGATA-*TGACT* |
| PAM2AG4AG | AAATATTTGAAAATTGTTTTTCACTAGATA-*TGAGT* |
